# A Robust 8-Gene Prognostic Signature for Early-Stage Non-small Cell Lung Cancer

**DOI:** 10.3389/fonc.2019.00693

**Published:** 2019-07-31

**Authors:** Ru He, Shuguang Zuo

**Affiliations:** ^1^Center for Translational Medicine, Huaihe Hospital of Henan University, Kaifeng, China; ^2^Institute of Infection and Immunity, Huaihe Hospital of Henan University, Kaifeng, China

**Keywords:** non-small cell lung cancer, overall survival, risk score, stage, prognostic signature

## Abstract

**Background:** The current staging system is imprecise for prognostic prediction of early-stage non–small cell lung cancer (NSCLC). This study aimed to develop a robust prognostic signature for early-stage NSCLC, allowing classification of patients with a high risk of poor outcome and specific treatment decision.

**Method:** In the present study, a comprehensive genome-wide profiling analysis was conducted using a retrospective pool of early-stage NSCLC patient data from the previous datasets of Gene Expression Omnibus (GEO) including GSE31210, GSE37745, and GSE50081 and The Cancer Genome Atlas (TCGA). Cox proportional hazards models were implemented to determine the association between gene expression levels and overall patient survival in each dataset. The common genes among all datasets were selected as candidate prognostic genes. A risk score model was developed and validated using four independent datasets and the entire cohort. The Kaplan-Meier with log-rank test was used to assess survival difference.

**Results:** A univariate Cox proportional hazards regression analysis for each dataset showed that a total of 2280 genes in GSE31210, 762 genes in GSE37745, 871 genes in GSE50081, and 666 genes in TCGA were identified as candidate protective genes, while overall 2131 genes in GSE31210, 913 in GSE37745, 1107 in GSE50081, and 997 in TCGA were identified as candidate risky genes. There were 8 common genes associated with overall survival, including 7 mRNA and 1 lncRNA. By using the Step-wise multivariate Cox analysis, an 8-gene prognostic signature (CDCP1, HMMR, TPX2, CIRBP, HLF, KBTBD7, SEC24B-AS1, and SH2B1) for early-stage NSCLC was developed. Patients in the high-risk group had shorter overall survival than those in the low-risk group. Multivariate regression and stratified analysis suggested that the prognostic power of the 8-gene signature was independent of other clinical factors. Furthermore, the 8-gene signature achieved AUC values of 0.726, 0.701, 0.725 and 0.650 in GSE31210, GSE37745, GSE50081 and TCGA, respectively. Moreover, the combination of the 8-gene signature and the stage resulted to a better patient classification for survival prediction and treatment decision.

**Conclusion:** This study developed a robust gene signature with great value for prognostic prediction in early-stage NSCLC, which may contribute to patient classification and personalized treatment decisions.

## Introduction

Lung cancer is a highly lethal malignant disease, the second most common cancer in men and women, and the leading cause of cancer-related death worldwide (1). Non-small cell lung cancer (NSCLC), accounts for 85% of all lung cancers, and is the predominant histological type. Despite recent therapeutic advances, patients with NSCLC are still associated with bleak outcomes, due to lack of early diagnostic and predictive biomarkers (2). Pulmonary resection is the primary treatment for early-stage NSCLC, with a 5-year survival rate of about 60% (3). Recently, it has been shown that adjuvant chemotherapy confers a survival advantage of 4–15% for patients with resected stage II–III (4–7), but not for patients with stage I disease (8, 9). The limited survival advantage suggests the deficiency of the current staging system and the presence of unknown tumor factors. It is imperative to develop novel prognostic biomarkers for risk stratification and treatment optimization in early patients.

Recent advances in microarray profiling and genome-wide sequencing have facilitated the identification of molecular prognostic factors that are crucial for precise classification of human cancers and personalized treatment decisions. A large number of studies in early-stage NSCLC have demonstrated that genomic data generated from patients with long-term follow-up are superior to the current staging system in estimating risk of worse prognosis. In those studies, numerous gene signatures have been generated to classify NSCLC patients with different clinical outcomes (10–14). However, no reliable and consistent gene signatures have emerged from these efforts. Additionally, the vast majority of studies have focused on single molecules, either mRNAs or lncRNAs (10, 15). Numerous works have demonstrated that mRNA and lncRNA signatures could precisely predict the prognosis of cancers (16–18). LncRNAs, a type of ncRNAs, have sequence lengths of more than 200 nucleotides with little or no protein-coding function (19), but mRNAs have protein-coding ability. LncRNAs and mRNAs crosstalk by sharing miRNA response elements, thereby generating competing endogenous RNA network (20). Relative to protein-coding mRNAs, lncRNAs are more closely associated with the status of cancer (21, 22). The single-biomarker for evaluating cancer prognosis is less robust relative to the more widely reported multiple-biomarker-based models (23). However, few studies have identified prognostic and predictive signatures by combining both mRNAs and lncRNAs. The increasing availability of genome-wide gene expression data in NSCLC makes it feasible to identify a robust gene signature. In the present study, several published datasets from the Gene Expression Omnibus (GEO) and The Cancer Genome Atlas (TCGA) were mined, in order to produce a robust prognostic signature for early-stage NSCLC. An 8-gene signature with reliable prognostic power in early-stage NSCLC was identified, which might cover the shortage of the current staging system, improve patient stratification, and provide promise for more personalized therapeutic interventions.

## Methods

### Patients and Study Design

The raw data of gene expression and corresponding clinical information of patients with early-stage NSCLC were downloaded from GEO and TCGA, respectively. In the study, three independent datasets were retrieved from GEO, including GSE31210 (24, 25), GSE37745 (26), and GSE50081 (27), and one dataset was employed from TCGA. After the samples without enough clinical information or with advanced disease were removed, a total of 1,331 patients were finally enrolled, including 226 patients from GSE31210, 165 from GSE37745, 181 from GSE50081, and 759 from TCGA. The gene expression data of the three GEO datasets were generated by Affymetrix U133 Plus 2.0 microarray platform, while the TCGA data were analyzed on the Illumina sequencing platform.

**Figure 1:**
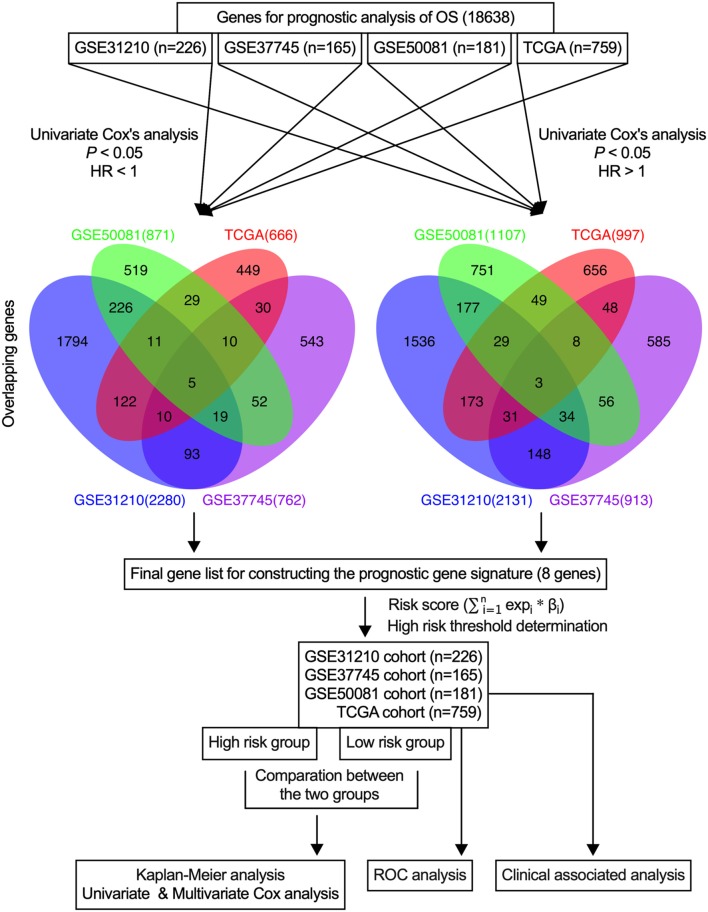
Study flow diagram. OS, overall survival.

In the present study, initially, the candidate genes that were associated with the overall survival of early-stage NSCLC patients from each dataset were identified, and the credible prognostic genes of the four overlapping datasets were selected. Then, the prognostic signature was developed using a risk score model and validated using four datasets and the entire cohort. Figure 1 illustrates the flow diagram of this study.

### Prognostic Signature

A univariate Cox proportional hazard regression model was used to assess the association of gene expression with the overall survival of NSCLC patients in each cohort. The hazard ratio (HR) from the univariate Cox regression analysis was used to identify candidate genes associated with the overall survival from each dataset. Genes with HR < 1 were considered as protective genes and those with HR > 1 were defined as risky genes. Meanwhile, genes with *P* < 0.05 were considered statistically significant. In order to improve reliability, only common genes between the four datasets were screened to construct the prognostic signature.

By combining the expression values of prognostic genes weighted by their regression coefficients, a risk score for each patient was constructed as follows:

$$\begin{array}{l} Risk\;score\;=\overset{n}{\underset{i=1}{\sum }}\exp_{i}^{*}\beta_{i} \end{array}$$

where *n* was the number of prognostic genes, exp_i_ the expression value of gene i, and β_i_ the regression coefficient of gene i in the univariate Cox regression analysis. Using the median risk score as a cutoff value, NSCLC patients were classified into high- and low-risk groups. Moreover, the relationship between the prognosis signature and disease-free survival was investigated based on the three cohorts of GSE31210, GSE37745, and GSE50081.

### Statistical Analysis

The Kaplan-Meier method was used to assess the differences in survival time of low- and high-risk NSCLC patients, and the log-rank test was used to determine the statistical significance of observed differences between groups. Multivariable Cox regression analysis and stratification analysis were used to assess whether the risk score was independent of other clinical features. The time-dependent receiver operating characteristic (ROC) curve was used to measure the prognostic performance by comparing the areas under the ROC curves (AUC). Significance was defined as *P* < 0.05.

## Results

### Prognostic Signature Generation

In this study, a univariate Cox proportional hazards regression analysis in each dataset was conducted, and candidate genes that were significantly correlated with the overall survival were identified. Under the cutoff values of *P* < 0.05 and HR < 1, 2,280 genes in GSE31210, 762 genes in GSE37745, 871 genes in GSE50081, and 666 genes in TCGA were identified as candidate protective genes. Under the cut-off values of *P* < 0.05 and HR > 1, 2,131 genes in GSE31210, 913 in GSE37745, 1,107 in GSE50081, and 997 in TCGA were identified as candidate risky genes. After combing the candidate protective genes in GSE31210, GSE37745, GSE50081, and TCGA, a total of 5 common genes were remained. Similarly, there were 3 common candidate risky genes after combing the identified candidate risky genes in the four datasets. By overlapping the four datasets, eight common genes (CDCP1, HMMR, TPX2, CIRBP, HLF, KBTBD7, SEC24B-AS1, and SH2B1) were finally identified, which were used to form the prognostic signature. The general information of the 8 genes is displayed in Table 1. Among them, 7 genes (CDCP1, HMMR, TPX2, CIRBP, HLF, KBTBD7, and SH2B1) were mRNA and one gene (SEC24B-AS1) was lncRNA. In Table 2, the prognostic correlation of the 8 genes with the overall survival of early-stage NSCLC patients in each dataset is shown.

**Table 1:** General information of the 8 genes for constructing the prognostic signature.

**Gene stable ID**	**Gene name**	**Gene type**	**Chromosome**	**Gene start (bp)**	**Gene end (bp)**
ENSG00000163814	CDCP1	Protein coding	3	45082278	45146422
ENSG00000099622	CIRBP	Protein coding	19	1259384	1274880
ENSG00000108924	HLF	Protein coding	17	55265012	55325065
ENSG00000072571	HMMR	Protein coding	5	163460203	163491945
ENSG00000120696	KBTBD7	Protein coding	13	41189833	41194566
ENSG00000247950	SEC24B-AS1	antisense	4	109347475	109433817
ENSG00000178188	SH2B1	Protein coding	16	28846600	28874212
ENSG00000088325	TPX2	Protein coding	20	31739271	31801805

**Table 2:** Univariate regression analysis of 8 genes and overall survival of NSCLC patients in 4 datasets.

**Genes**	**GSE31210**		**GSE37745**		**GSE5008**		**TCGA**	
	**HR (95% CI)**	***P***	**HR (95% CI)**	***P***	**HR (95% CI)**	***P***	**HR (95% CI)**	***P***
CDCP1	2.17 (1.28–3.67)	3.80E-03	1.93 (1.42–2.63)	2.40E-05	1.42 (1.04–1.95)	3.00E-02	1.17 (1.02–1.35)	2.20E-02
CIRBP	0.25 (0.13–0.48)	3.00E-05	0.65 (0.45–0.95)	2.70E-02	0.6 (0.39–0.93)	2.30E-02	0.67 (0.53–0.85)	8.40E-04
HLF	0.7 (0.57–0.86)	6.40E-04	0.85 (0.74–0.99)	3.40E-02	0.76 (0.63–0.92)	5.80E-03	0.92 (0.85–0.99)	3.10E-02
HMMR	1.61 (1.21–2.13)	1.00E-03	1.35 (1.1–1.66)	3.80E-03	1.29 (1.02–1.63)	3.50E-02	1.16 (1.01–1.33)	3.60E-02
KBTBD7	0.39 (0.18–0.83)	1.60E-02	0.71 (0.52–0.97)	3.20E-02	0.64 (0.46–0.9)	9.60E-03	0.77 (0.61–0.97)	2.40E-02
SEC24B-AS1	0.3 (0.17–0.55)	9.90E-05	0.62 (0.4–0.97)	3.70E-02	0.48 (0.24–0.95)	3.50E-02	0.78 (0.65–0.94)	1.00E-02
SH2B1	0.34 (0.15–0.79)	1.20E-02	0.38 (0.19–0.74)	4.70E-03	0.32 (0.13–0.78)	1.30E-02	0.79 (0.65–0.96)	1.70E-02
TPX2	1.49 (1.18–1.89)	8.90E-04	1.17 (1.01–1.35)	3.70E-02	1.27 (1.08–1.49)	4.40E-03	1.12 (1–1.24)	5.00E-02

*HR, hazard ratio; CI, confidence interval*.

### 8-Gene Prognostic Signature Validation

**Figure 2:**
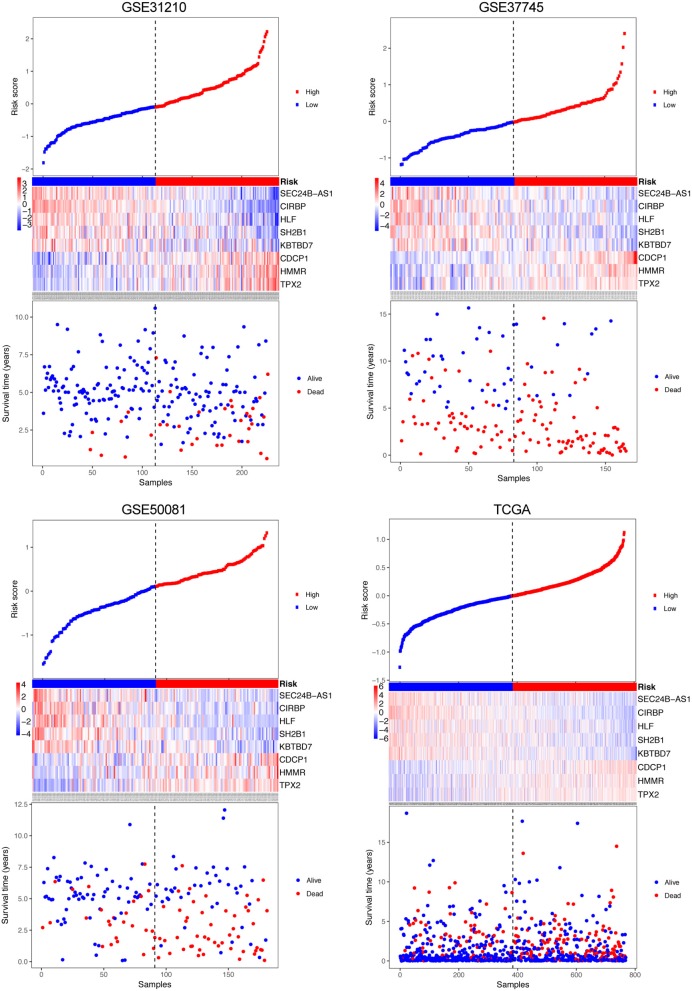
Risk-score analysis of early-stage NSCLC patients in the four datasets. In each dataset, the risk score distribution, gene expression profiles, and patients' survival status are displayed. The black-dotted line represents the median cut-off, dividing patients into high- and low-risk groups.

**Figure 3:**
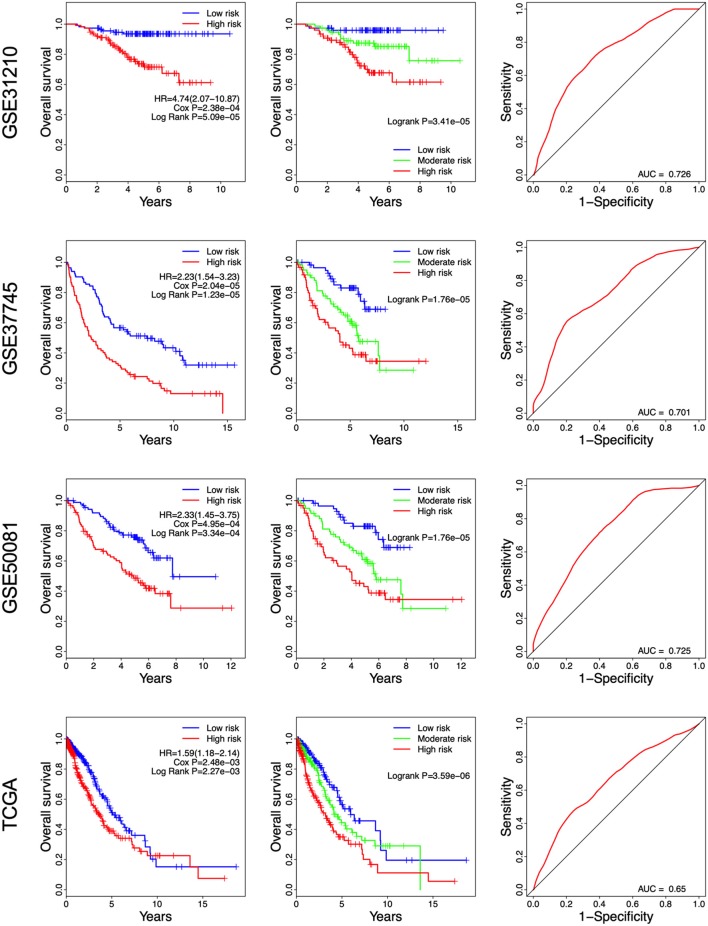
Kaplan-Meier and ROC curves for the 8-gene signature in the four datasets. Patients with high risk scores had poor outcome in terms of overall survival.

A risk score was constructed with the regression coefficients from the univariate Cox analysis, and a prognostic model was developed to predict overall survival. In the prognostic model, the risk score for each patient was calculated. The patients in each dataset were classified into high- and low-risk groups, based on the median risk score, which was used as the cutoff point. In Figure 2, the risk score distribution, gene expression, and the patients' survival status in each dataset were displayed, ranked according to the risk score values for the 8-gene signature. The resulted data demonstrated that the patients in the high-risk group had a shorter overall survival than those in the low-risk group (GSE31210: HR = 4.74, 95% CI = 2.07–10.87, *P* = 5.09e-05; GSE37745: HR = 2.23, 95% CI = 1.54–3.23, *P* = 1.23e-05; GSE50081: HR = 2.33, 95% CI = 1.45–3.75, *P* = 3.34e-04; TCGA: HR = 1.59, 95% CI = 1.18–2.14, *P* = 2.25e-03) (Figure 3 Left panel). Then, we evaluated the survival difference in 3 groups, including high-, moderate-, and low-risk groups. The results showed that the higher the risk score was, the worse the survival of patients was (GSE31210: *P* = 3.41e-05; GSE37745: *P* = 1.76e-05; GSE50081: *P* = 1.76e-05; TCGA: *P* = 3.50e-06) (Figure 3 Middle panel). These results confirmed that risk score can be used as a prognostic indicator. The time-dependent ROC curves showed that the 8-gene signature achieved AUC values of 0.726, 0.701, 0.725, and 0.650 in GSE31210, GSE37745, GSE50081, and TCGA, respectively (Figure 3 Right panel), suggesting a substantially effective performance for overall survival prediction.

**Figure 4:**
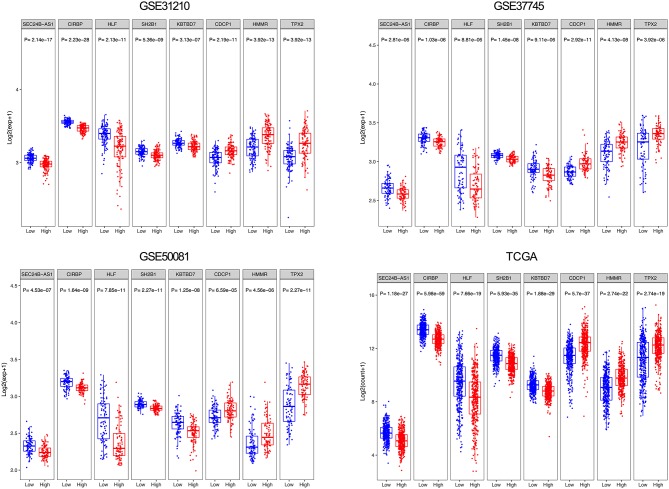
Box plot visualization of the expression levels of the 8 genes in the risk groups.

From the eight genes, 3 were associated with high risk (CDCP1, HMMR, and TPX2; HR > 1) and 5 appeared to be protective (CIRBP, HLF, KBTBD7, SEC24B-AS1, and SH2B1; HR < 1). The expression of the 8 prognostic genes was detected and the differences between high- and low-risk groups were compared. Patients with high-risk scores tended to express risky genes, whereas patients in the low-risk group tended to express protective genes (Figure 4).

### The 8-Gene Prognostic Signature Is Independent of Other Clinicopathological Factors

In order to evaluate the contribution of the 8-gene signature as an independent prognostic factor of patient survival, a multivariate Cox regression analysis was performed using a stepwise method. Covariates included the gene signature and clinicopathological factors, such as age, gender, stage, histologic type, gene mutation, smoking, and performance status. The results showed that the predictive ability of the 8-gene signature was independent of other clinicopathological factors for overall survival of early-stage NSCLC patients in four independent datasets (GSE31210: HR = 3.51, 95% CI = 1.47–8.37, *P* = 4.60E-03; GSE37745: HR = 2.69, 95% CI = 1.82–3.97, *P* = 6.00E-07; GSE50081: HR = 1.92, 95% CI = 1.10–3.37, *P* = 2.20E-02; TCGA: HR = 1.47, 95% CI = 1.08–2.00, *P* = 1.40E-02) (Table 3) and the entire cohort (HR = 1.90, 95% CI = 1.55–2.33, *P* = 8.70E-10) (Table 4). Stage IB is the indication of adjuvant chemotherapy. Univariate and multivariate Cox regression model suggested that stage IA/IB in GSE37745 (HR = 1.71, 95% CI = 1.07–2.73, *P* = 2.50E-02 for univariate model, and HR = 1.69, 95% CI = 1.04–2.72, *P* = 3.30E-02 for multivariate model) was significantly correlated with overall survival of the patients, but stage IA/IB in other three cohorts did not show any significant association with overall survival (Table 3).

**Table 3:** Univariate and multivariate Cox regression analyses of the gene signature and overall survival of NSCLC patients in 4 independent datasets.

**Variables**		**Patients (*N*)**	**Univariate analysis**		**Multivariate analysis**	
			**HR (95% CI)**	***P***	**HR (95% CI)**	***P***
**GSE31210**						
Age	< = 65/>65	176/50	2.58 (1.31–5.08)	6.00E-03	2.71 (1.31–5.63)	7.50E-03
Gender	Male/Female	121/105	1.52 (0.78–2.96)	2.20E-01		
ALK fusion	−/+	215/11	1.49 (0.36–6.24)	5.80E-01		
EGFR mutation	−/+	99/127	0.47 (0.24–0.93)	3.00E-02	0.77 (0.36–1.67)	5.10E-01
KRAS mutation	−/+	206/20	0.87 (0.27–2.85)	8.20E-01		
Myc	Low/High	207/17	0.70 (0.17–2.90)	6.20E-01		
Stage	IA/IB	114/54	2.47 (0.95–6.40)	6.30E-02	1.69 (0.64–4.46)	2.90E-01
Stage	IA/II	114/58	6.18 (2.68–14.26)	1.90E-05	4.89 (2.00–11.95)	4.90E-04
Smoking	No/Yes	115/111	1.64 (0.84–3.20)	1.50E-01		
Risk score	Low/High	113/113	4.74 (2.07–10.87)	2.40E-04	3.51 (1.47–8.37)	4.60E-03
**GSE37745**						
Age	< = 65/>65	82/83	1.51 (1.05–2.18)	2.60E-02	1.46 (1.00–2.13)	4.80E-02
Gender	Male/Female	74/91	1.11 (0.77–1.60)	5.90E-01		
Stage	IA/IB	40/90	1.71 (1.07–2.73)	2.50E-02	1.69 (1.04–2.72)	3.30E-02
Stage	IA/II	40/35	1.77 (1.02–3.06)	4.30E-02	1.81 (1.01–3.23)	4.50E-02
Histological type	Adeno/Large	89/21	0.97 (0.54–1.73)	9.10E-01		
Histological type	Adeno/Squamous	89/55	1.25 (0.84–1.85)	2.80E-01		
Performance status	0/1	87/66	2.04 (1.39–2.98)	2.40E-04	2.26 (1.53–3.34)	4.10E-05
Performance status	0/2–3	87/12	1.50 (0.74–3.04)	2.60E-01	1.47 (0.70–3.08)	3.10E-01
Risk score	Low/High	83/82	2.23 (1.54–3.23)	2.00E-05	2.69 (1.82–3.97)	6.00E-07
**GSE50081**						
Age	< = 65/>65	59/122	1.56 (0.93–2.61)	9.00E-02	1.30 (0.74–2.30)	3.60E-01
Gender	Male/Female	83/98	1.93 (1.19–3.14)	7.80E-03	1.73 (0.99–3.00)	5.30E-02
Stage	IA/IB	48/79	1.76 (0.93–3.34)	8.20E-02	1.78 (0.83–3.83)	1.40E-01
Stage	IA/II	48/54	2.46 (1.27–4.78)	7.80E-03	3.13 (1.39–7.01)	5.70E-03
Histological type	Adeno/Large	127/7	1.71 (0.62–4.74)	3.00E-01		
Histological type	Adeno/Other	127/4	1.84 (0.57–5.92)	3.00E-01		
Histological type	Adeno/Squamous	127/43	0.8 (0.46–1.39)	4.30E-01		
Smoking	No/Yes	24/136	1.39 (0.66–2.92)	3.90E-01		
Risk score	Low/High	91/90	2.33 (1.45–3.75)	4.90E-04	1.92 (1.10–3.37)	2.20E-02
**TCGA**						
Age	< = 65/>65	325/434	1.29 (0.95–1.75)	1.10E-01		
Gender	Male/Female	306/453	0.96 (0.71–1.29)	7.70E-01		
Stage	IA/IB	213/277	1.31 (0.87–1.97)	1.90E-01	1.23 (0.82–1.85)	3.20E-01
Stage	IA/II	213/269	2.04 (1.36–3.05)	5.30E-04	1.94 (1.28–2.92)	1.60E-03
Histological type	Adeno/Squamous	372/387	1.18 (0.88–1.60)	2.70E-01		
Risk score	Low/High	380/379	1.59 (1.18–2.14)	2.50E-03	1.47 (1.08–2.00)	1.40E-02

*HR, hazard ratio; CI, confidence interval; Adeno, adenocarcinoma; Large, large cell carcinoma; Squamous, squamous cell carcinoma*.

**Table 4:** Univariate and multivariate Cox regression analyses of the gene signature and overall survival of NSCLC patients in entire cohort.

**Variables**		**Patients (*N*)**	**Univariate analysis**		**Multivariate analysis**	
			**HR (95% CI)**	***P***	**HR (95% CI)**	***P***
Age	< = 65/>65	646/693	1.85 (1.51–2.25)	1.40E-09	1.77 (1.44–2.17)	4.00E-08
Gender	Male/Female	591/749	1.34 (1.10–1.63)	3.90E-03	1.11 (0.91–1.37)	3.00E-01
Stage	I/II	923/417	1.75 (1.43–2.14)	5.80E-08	1.62 (1.32–1.99)	3.80E-06
Histological type	Adeno/Large	819/28	1.61 (1.00–2.62)	5.20E-02	1.53 (0.94–2.49)	8.70E-02
Histological type	Adeno/Other	819/4	2.10 (0.67–6.57)	2.00E-01	1.83 (0.58–5.76)	3.00E-01
Histological type	Adeno/Squamous	819/489	1.76 (1.44–2.15)	3.00E-08	1.33 (1.08–1.64)	7.80E-03
Risk score	Low/High	671/669	2.07 (1.69–2.53)	1.60E-12	1.90 (1.55–2.33)	8.70E-10

*HR, hazard ratio; CI, confidence interval; Adeno, adenocarcinoma; Large, large cell carcinoma; Squamous, squamous cell carcinoma*.

### Stratification Analysis

**Figure 5:**
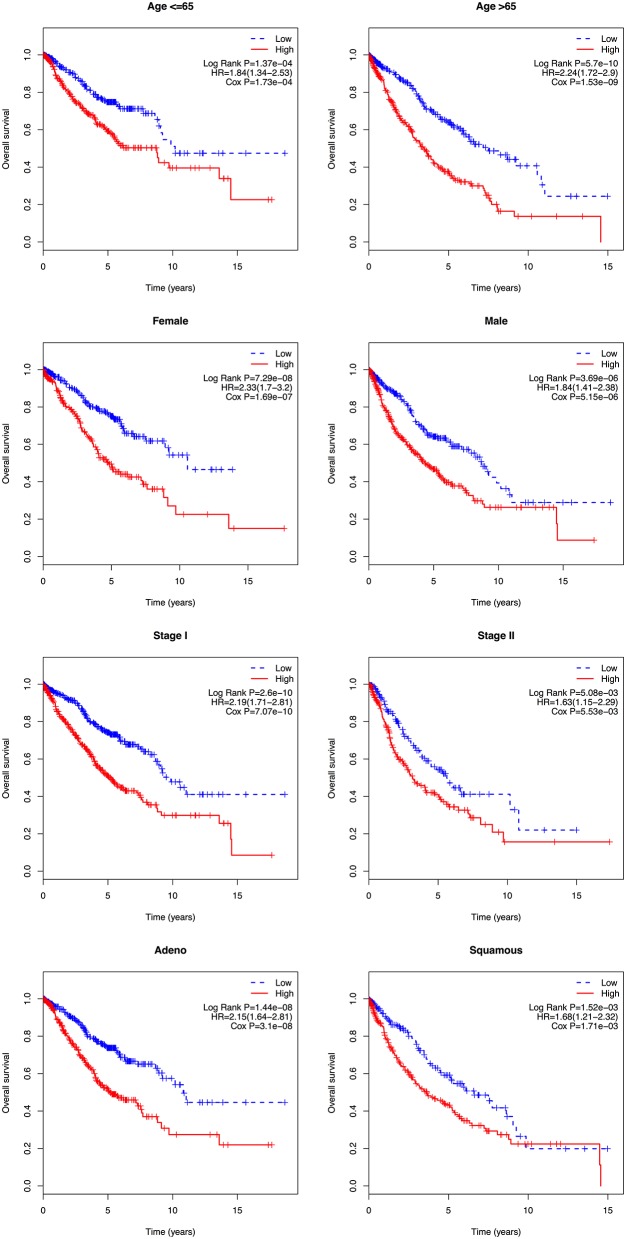
Kaplan-Meier analysis of overall survival for patients stratified by age, gender, stage, and histological type.

In the multivariate Cox regression analysis, several clinicopathological factors were also identified as independent prognostic factors. Subsequently, a stratification analysis was carried out to evaluate whether the 8-gene signature could predict patient survival within the same clinical factor subgroup. Patients in the entire cohort were factitiously stratified based on clinical parameters, such as age ( ≤ 65/>65), gender (female/male), stage (I/II), and histologic type (adeno/squamous). The results showed that the 8-gene signature could classify patients of the same stratum of age, gender, stage, and histologic type into high- and low-risk groups. Patients with high risk scores had a shorter overall survival than those with low risk scores in each stratum (Figure 5).

### Survival Prediction by Stage and 8-Gene Signature Combination

**Figure 6:**
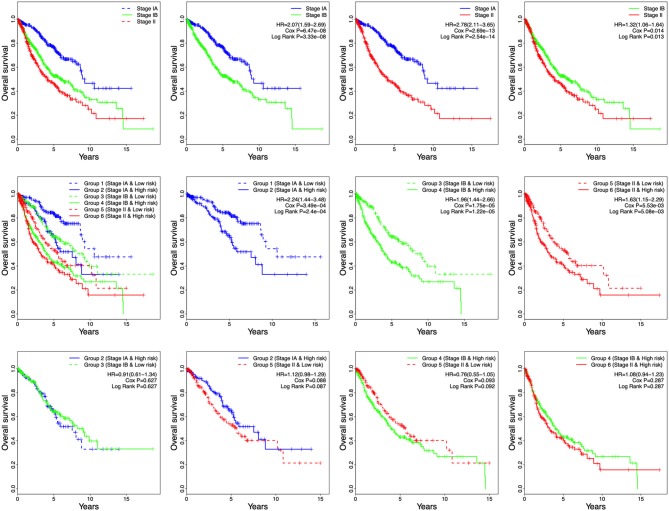
Kaplan-Meier analysis of overall survival for patients grouped by stage and 8-gene signature combination.

Tumor stage has great survival predictive value in clinical practice. In this study, stage and risk score were proven to be independent prognostic factors in all four independent datasets and the entire cohort. Therefore, the development of a prognostic model for survival prediction was attempted by combining the stage with the 8-gene signature. Based on the stage status and the risk score, patients were divided into six groups: Group 1 (Stage IA and Low risk), Group 2 (Stage IA and High risk), Group 3 (Stage IB and Low risk), Group 4 (Stage IB and High risk), Group 5 (Stage II and Low risk), and Group 6 (Stage II and High risk) (Figure 6). Based on the results shown in Figure 6, the patients in each stage were all classified into low- and high-risk groups, and the patients of each stage in high-risk group had poor prognosis. The results indicated the patients in Group 2 had worse outcomes than those in Group 1, Group 4 had worse outcomes than those in Group 3, and Group 6 had worse outcomes than those in Group 5 (Figure 6). However, there was no significant difference in overall survival between the patients in Group 2 and Group 3/5. Furthermore, no difference in overall survival was observed between Group 4 and Group 5/6 (Figure 6). These results suggest that patients with high risk score in stage IA might have similar prognosis as those with low risk score in stage IB and stage II, suggesting that adjuvant chemotherapy should also be used in patients with stage IA who have a high risk score.

Among the six groups, Group 1 showed the best prognosis, whereas Group 6 exhibited the worst. In future practice, patients could be classified into six groups according to their stages and risk scores to predict clinical outcomes. Significantly, there was difference in overall survival between stage IA and IB in the combined dataset (Figure 6, HR = 2.07, 95% CI = 1.59–2.69, *P* = 3.33e-08).

### Relationship Between the Prognosis Signature and Disease-Free Survival

**Figure 7:**
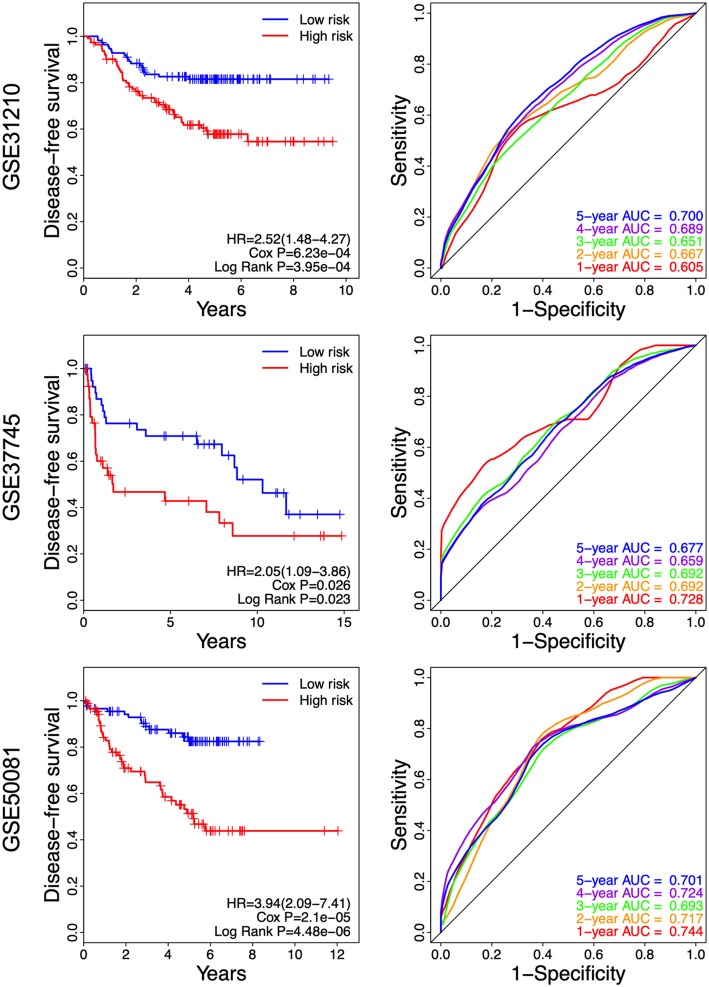
Kaplan-Meier and ROC curves for the 8-gene signature in the three datasets. Patients in the high risk groups had shorter disease-free survival than those in the low-risk groups.

As shown in Figure 7, we found that the NSCLC patients in the high-risk group had a shorter disease-free survival, compared with those in the low-risk group (GSE31210: HR = 2.52, 95% CI = 1.48–4.27, *P* = 3.95e-04; GSE37745: HR = 2.05, 95% CI = 1.09–3.86, *P* = 0.023; and GSE50081: HR = 3.94, 95% CI = 2.09–37.41, *P* = 4.48e-06. Higher AUC stands for a better performance. The time-dependent ROC curves showed that the AUC for the 8-gene signature achieved 0.605, 0.667, 0.651, 0.689, and 0.700 for the 1, 2, 3, 4, and 5 year survival in GSE31210, respectively. The 8-gene signature obtained 0.728, 0.692, 0.692, 0.659, and 0.677 for the 1, 2, 3, 4, and 5 year survival in GSE37745. Moreover, the AUC values of the 8-gene signature for the 1, 2, 3, 4, and 5 year survival in GSE50081 were respectively 0.744, 0.717, 0.693, 0.724, and 0.701 (Figure 7). These results suggest that there is a substantially effective performance for predicting disease-free survival.

## Discussion

Increased understanding of the genomic changes of early-stage NSCLC promotes the discovery of prognostic and predictive signatures, and allows personalized treatment decisions. In this study, a novel 8-gene prognostic signature using genome-wide expression data from early-stage NSCLC patients was developed and validated. The developed 8-gene signature was able to identify early-stage NSCLC patients with high and low risk for poor prognosis. This signature may constitute an important step forward in treatment decision for early-stage NSCLC patients.

Previous studies have identified many molecular signatures that classify patients into different prognostic groups (10–14). However, these putative prognostic signatures demonstrated minimal overlap (10, 28). The discordant findings have been attributed to insufficient sample size, biological heterogeneity, various expression platforms, and different statistical methods (10). In general, studies often use a training set to develop prognostic signatures (10, 12), which might lead to the discordance. In the present study, survival-related genes were identified using a large patient cohort of four independent datasets. Only the common among the four datasets genes were selected to build the gene signature, providing a more robust and reliable signature, relative to that derived from a single dataset, and partially handling the problem of discordance.

The mRNA CDCP1 was one of the 8-gene prognostic signature in our study. CDCP1, as a transmembrane protein, has been demonstrated to express in stem cells as well as hematopoietic cells (29). CDCP1 has been implicated to be highly expressed in many kinds of cancer cells, and to be related to over-proliferation, migration, invasion, and lymph node metastasis of lung cancer (30–32). Moreover, CDCP1 up-regulation is associated with the worse overall survival and recurrence-free survival of cancers (32, 33). HMMR has been demonstrated to inhibit cell proliferation of glioma in a dose-dependent way (34). HAMM over-expression in cancers has been implicated to cause centrosomal and mitotic dys-regulation, and to mediate apoptosis as well as cell cycle pathways (35). Moreover, HAMM has been suggested to have prognostic value, and affect the proliferative ability of chronic lymphocytic leukemia cells (36). Increased HAMM is correlated with poor prognosis in aggressive cancers (37, 38). A former study has reported that there is a positive correlation between TPX2 up-regulation and lymph node metastasis, TNM stage, as well as poor prognosis of patients in cancers including cholangiocarcinoma, gastric cancer, and lung adenocarcinoma (39–41). Moreover, TPX2 silence resulted in G2-M arrest, apoptosis and the suppression of cell migration and invasion of cancers (39, 42). TPX2 has been documented to mediate the cell growth and apoptosis via regulating PI3K/AKT/P21 signaling pathway in breast cancer (43). CIRBP high expression has been documented to have significantly better 5 year disease-free survival rate (44). CIRBP has been suggested to be a potential cell cycle regulator, and the loss of CIRBP might participate in the progression of endometrial carcinogenesis (45). Waters et al. (46) have suggested that HLF regulates the cell death, and is abnormally expressed in cancers. Chen et al. (47) have demonstrated that HLF-mediated miR-132 directly inhibits the expression of TTK, thereby playing inhibitory effects on cell growth, metastasis, as well as radio resistance of glioma. SH2B1, one member of the SH2B family, has been documented to serve as tumor activators in cancers. A previous study has implicated that SH2B1 is highly-expressed and linked with epithelial to mesenchymal transition biomarkers and poor prognosis in patients with lung adenocarcinoma, and SH2B1 has important roles on cell proliferation, migration, and invasion in A549 and H1299 cells (48). SH2B1 has been reported to be highly expressed in NSCLC tissues and cells, and SH2B1 high-expression has poor disease-free survival and overall survival (49). KBTBD7 has been found to be involved in inflammation and cardiac dysfunction, which is targeted by miR-21 (50). However, the roles of KBTBD7 and SEC24B-AS1 in cancer have not been investigated. Of note, the result of the AUC analyses in our study showed that the AUC values of the combination of 8 genes were more than 0.60 in both the overall survival and disease-free survival, suggesting that the combination of 8 genes could be regarded as a novel factor that may serve as a prognosis indicator for NSCLC patients. Stratification analysis indicated that the 8-gene signature predicted survival in most sub-groups and was independent of other clinical factors, such as age, gender, stage, and histology type. In our study, the 8-gene signature showed a great ability to stratify NSCLC patients into high- and low-risk groups with significantly different overall survival. Thus, it could be an important asset in improving the prognosis and providing better prescriptions.

Currently, the tumor staging system remains the most powerful tool for survival prediction and treatment decision in NSCLC patients (51). Despite its great clinical value, its prognostic and predictive power is incompetent to guide patient management. In particular, the current staging system is far from accurate in predicting survival at the individual level, since half of the patients with early disease will eventually develop recurrent disease (51). This is directly linked to the decision of prescribing adjuvant chemotherapy after a pulmonary resection in early-stage NSCLC patients. Identifying early-stage patients with poor prognosis would consequently help specialists screen the appropriate candidates for adjuvant chemotherapy. Further development of genomic signatures is expected to assist patient stratification in clinical practice. In the present stratification analysis, the 8-gene signature showed prognostic value among stage IA, stage IB and stage II patients. It was able to classify patients in the same stage into high- and low-risk groups with significantly different survival prospects, implying that the 8-gene signature can improve the accuracy of survival prediction. In addition, a prognostic model was developed by combining the stage with the 8-gene signature for survival prediction. These findings might help specialists select high-risk patients for adjuvant therapy in addition to surgery resection.

Significantly, in our study, univariate and multivariate Cox regression model suggested that stage IA/IB in GSE37745 was significantly correlated with overall survival of the patients. Moreover, the patients in stage IB had worse overall survival than those in stage IA in the combined dataset. Strauss et al. have demonstrated that adjuvant chemotherapy is not standard care for stage IB NSCLC patients (52). However, another previous study has demonstrated that there is a remarkable survival improvement in stage IB NSCLC patients from Italy treated with adjuvant chemotherapy (53). These results that adjuvant chemotherapy is efficient for stage IB NSCLC patients with large tumors (51, 54).

These findings may have substantial clinical value for NSCLC. Remarkably, several limitations should be noted in our study. Firstly, data of ALK/EGFR/KRAS was only available in GSE31210, and there were no data of molecular status in the rest of the cohorts, thus, there was insufficient sample size to assess an association or not with the 8-gene signature. Secondly, our study was the retrospective nature of the research and had the heterogeneity of the techniques that have been used to analyze gene expression (Affymetrix U133 Plus 2.0 microarray platform and different Illumina sequencing platform). Thirdly, further studies should be carried out to determine the biological roles of these predictive mRNAs and lncRNAs relying on *in vitro* and *in vivo* data based on all kinds of experiment methods.

## Conclusions

A novel 8-gene signature for prognostic prediction in early-stage NSCLC patients was developed. The findings suggested that the 8-gene signature is a powerful predictor for overall survival of patients with early-stage NSCLC. Furthermore, the signature was independent of other clinical factors, such as stage. Additionally, a prognostic model combining the 8-gene signature with the stage was developed, which may conduce to treatment decisions for individuals and hold promise for clinical practice in the near future.

## Data Availability

The datasets used and/or analyzed during the current study are available from the corresponding author on reasonable request.

## Author Contributions

RH: download the data and wrote the manuscript. SZ: conceived and designed the study, performed the analysis, and contributed to critical review of the manuscript. All authors read and approved the final manuscript.

### Conflict of Interest Statement

The authors declare that the research was conducted in the absence of any commercial or financial relationships that could be construed as a potential conflict of interest.
